# Refractory Ileal Perforations in a Cytomegalovirus-Infected Premature Neonate Resolved After Ganciclovir Therapy

**DOI:** 10.3389/fped.2020.00352

**Published:** 2020-07-14

**Authors:** Mari Morimoto, Hirofumi Sawada, Noriko Yodoya, Hiroyuki Ohashi, Kuniaki Toriyabe, Ryo Hanaki, Katsumi Sugiura, Hidemi Toyoda, Kohei Matsushita, Yuhki Koike, Kohei Otake, Mikihiro Inoue, Keiichi Uchida, Hiroshi Imai, Yoshihide Mitani, Kazuo Maruyama, Yoshihiro Komada, Tomoaki Ikeda, Masahiro Hirayama

**Affiliations:** ^1^Department of Pediatrics, Mie University Graduate School of Medicine, Tsu, Japan; ^2^Anesthesiology and Critical Care Medicine, Mie University Graduate School of Medicine, Tsu, Japan; ^3^Obstetrics and Gynecology, Mie University Graduate School of Medicine, Tsu, Japan; ^4^Department of Pediatric Surgery, Mie University Graduate School of Medicine, Tsu, Japan; ^5^Department of Pediatric Surgery, Mie Prefectural General Medical Center, Yokkaichi, Japan; ^6^Pathology, Mie University Graduate School of Medicine, Tsu, Japan

**Keywords:** cytomegalovirus, congenital viral infection, ganciclovir, gastrointestinal perforation, pathological examination, premature neonate, necrotizing enterocolitis, spontaneous intestinal perforation

## Abstract

Severe neonatal gastrointestinal diseases such as necrotizing enterocolitis or spontaneous intestinal perforation are potentially lethal conditions which predominantly occur in preterm infants. Cytomegalovirus (CMV), which is known to cause congenital and acquired infections in the newborns, has also been implicated in such severe gastrointestinal diseases in premature infants. However, the pathogenic role of CMV and effect of antiviral therapy in severe gastrointestinal disease in premature neonates is currently unclear. We present an infant, born at 26-weeks' gestation, presented with progressive dyspepsia and abdominal distention after the closure of the symptomatic patent ductus arteriosus at the day of life (DOL) 4, requiring the emergent surgery for ileal perforation at the DOL8. After the surgery, abdominal symptoms persisted and the second emergent surgery was performed for the recurrent ileal perforation at DOL17. Even then the abdominal symptoms prolonged and pathological examination in the affected intestine at the second surgery showed CMV inclusion body. Immunoreactivity for CMV antigen was detected in the specimen at the first surgery on DOL8. Blood and urinary CMV-DNA were detected at DOL28. CMV-DNA was also detected in the dried umbilical cord which was obtained within a week from birth. A 6-week course of intravenous ganciclovir (12 mg/kg/day) was started at DOL34 and then symptoms resolved along with decreasing blood CMV-DNA. Pathological findings characteristic of CMV were not detected in the resection specimen at the ileostomy closure at DOL94. These observations indicate that anti-CMV therapy may be beneficial for some premature infants with severe CMV-associated gastrointestinal diseases and warrants further studies focusing on pathogenic role, diagnosis, treatment and prevention of this underrecognized etiology of severe gastrointestinal diseases particularly in premature neonates.

## Introduction

Necrotizing enterocolitis (NEC) or spontaneous intestinal perforation (SIP), which predominantly occur in preterm infants, increase the risk of mortality and morbidity and are associated with adverse neurodevelopmental outcome in the affected infants (1). Cytomegalovirus (CMV) causes congenital and acquired infections in newborns. Approximately 5–10% of the newborns with congenital CMV infection cause perinatal disease including symptoms such as rash, jaundice and microcephaly which may lead to audiological, ophthalmological and neurological sequelae (2). Perinatal or postnatal CMV infection through infected maternal genital tract or breast milk also cause symptomatic disease in premature infants (3–5). Over the past decades, neonates with intestinal disease (e.g., NEC, SIP, stricture, ulceration, volvulus and abdominal compartment syndrome) associated with CMV infection, regardless of the route of viral transmission, have been described in several case reports and case series (6–8). Although a 6-weeks of ganciclovir (GCV) therapy has been associated with improved neurodevelopmental outcomes for the infants with congenital CMV infection (9), pathogenic relevance of CMV or benefit of GCV therapy in such CMV-associated intestinal diseases in neonates are not fully elucidated.

## Case Report

**Figure 1:**
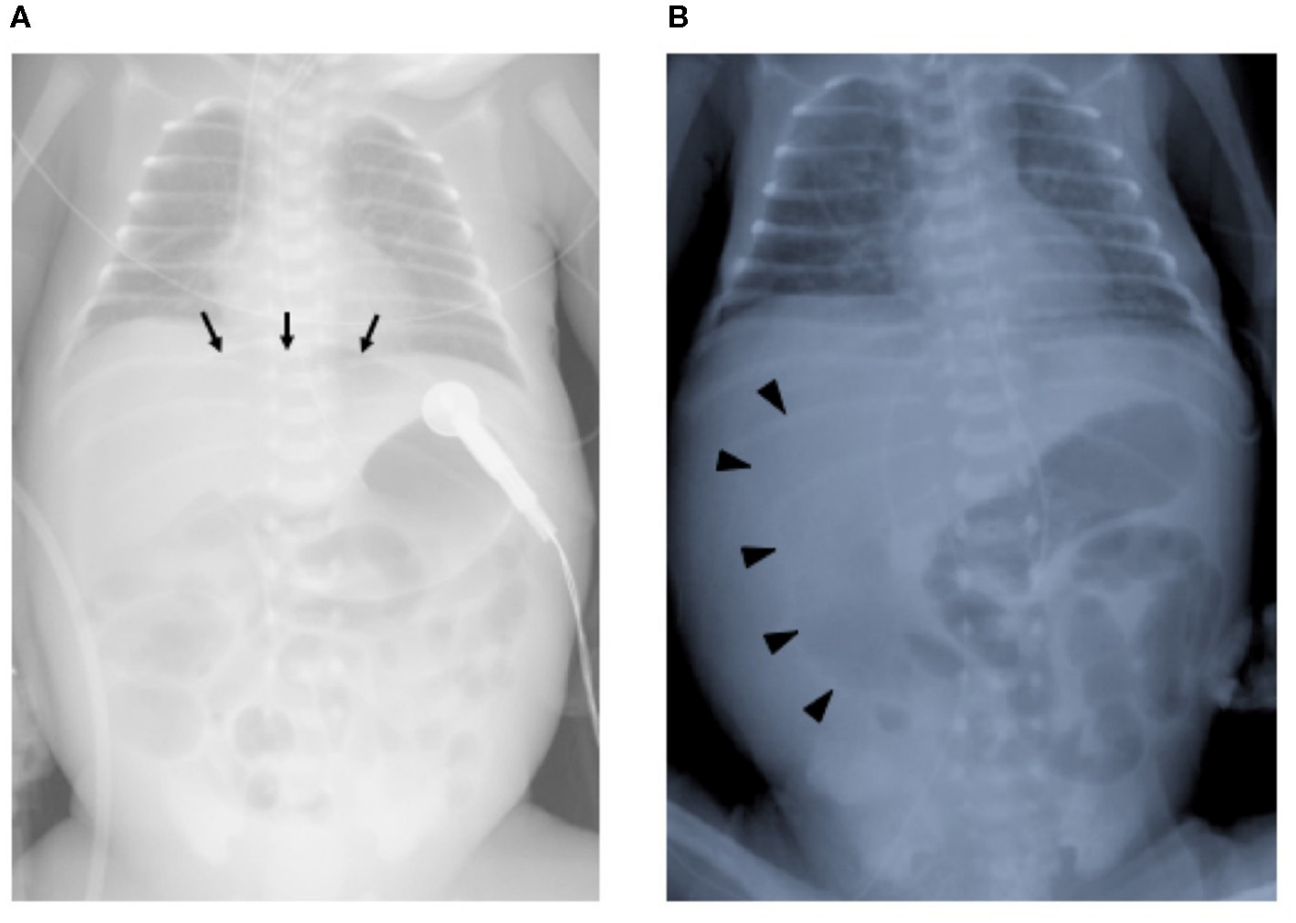
Chest and abdominal radiographs at the ileal perforations. **(A)** First perforation at day of life 8. Arrows indicate free air in the intraperitoneal space. **(B)** Second perforation at day of life 17. Arrowheads indicate free air in the intraperitoneal space.

**Figure 2:**
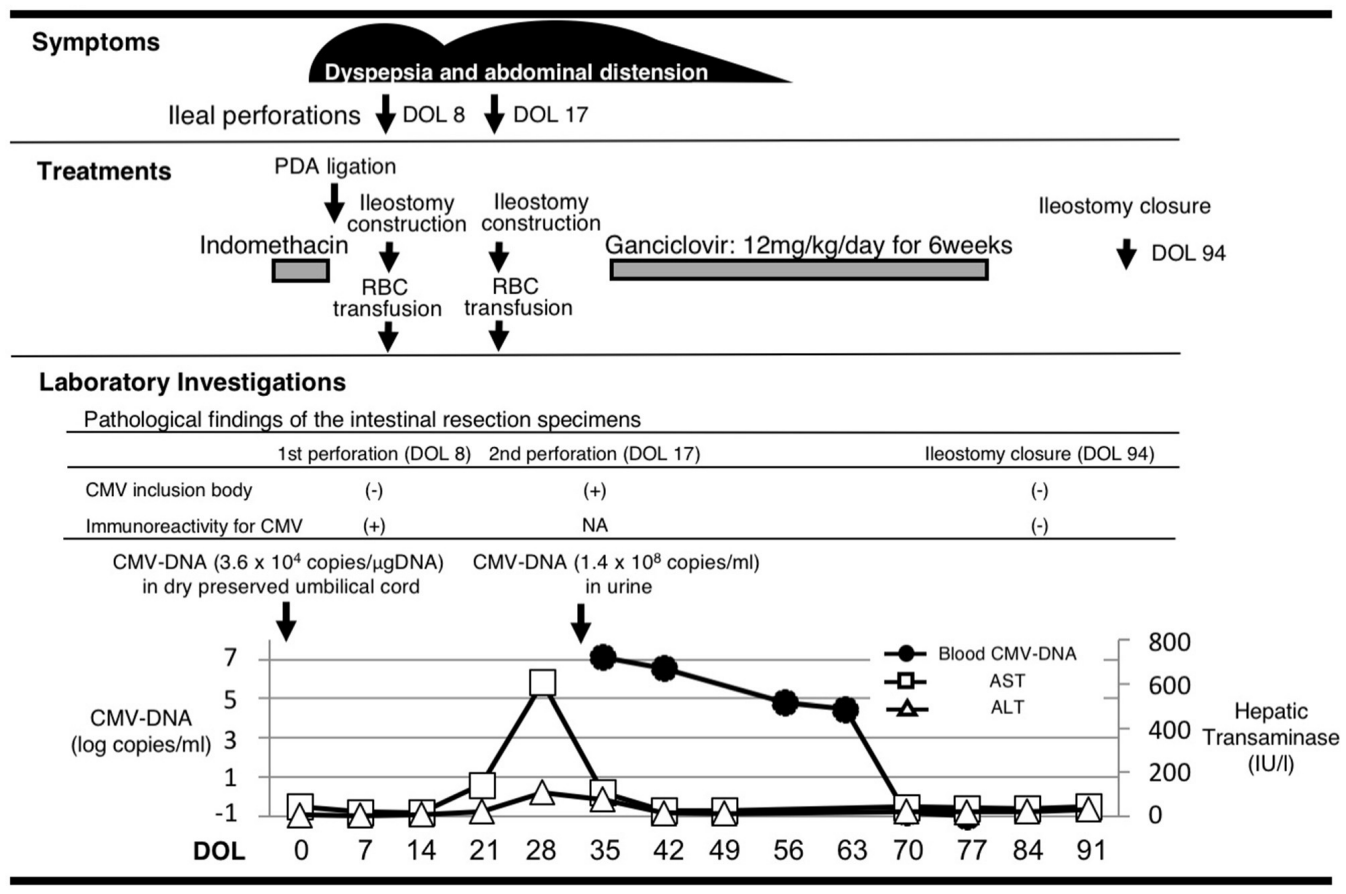
The association of clinical manifestation and treatments with the laboratory findings. Histological analyses of the affected intestines were performed before and after the 6-week treatment with ganciclovir. Cytomegalovirus DNA was measured in the dry preserved umbilical cord obtained within a week from birth, the urine and the blood at the designated time points by the real-time polymerase chain reaction assay. DOL, day of life; PDA, patent ductus arteriosus; RBC, red blood cell; CMV, cytomegalovirus; NA, not available; AST, aspartate aminotransferase; ALT, alanine aminotransferase.

**Figure 3:**
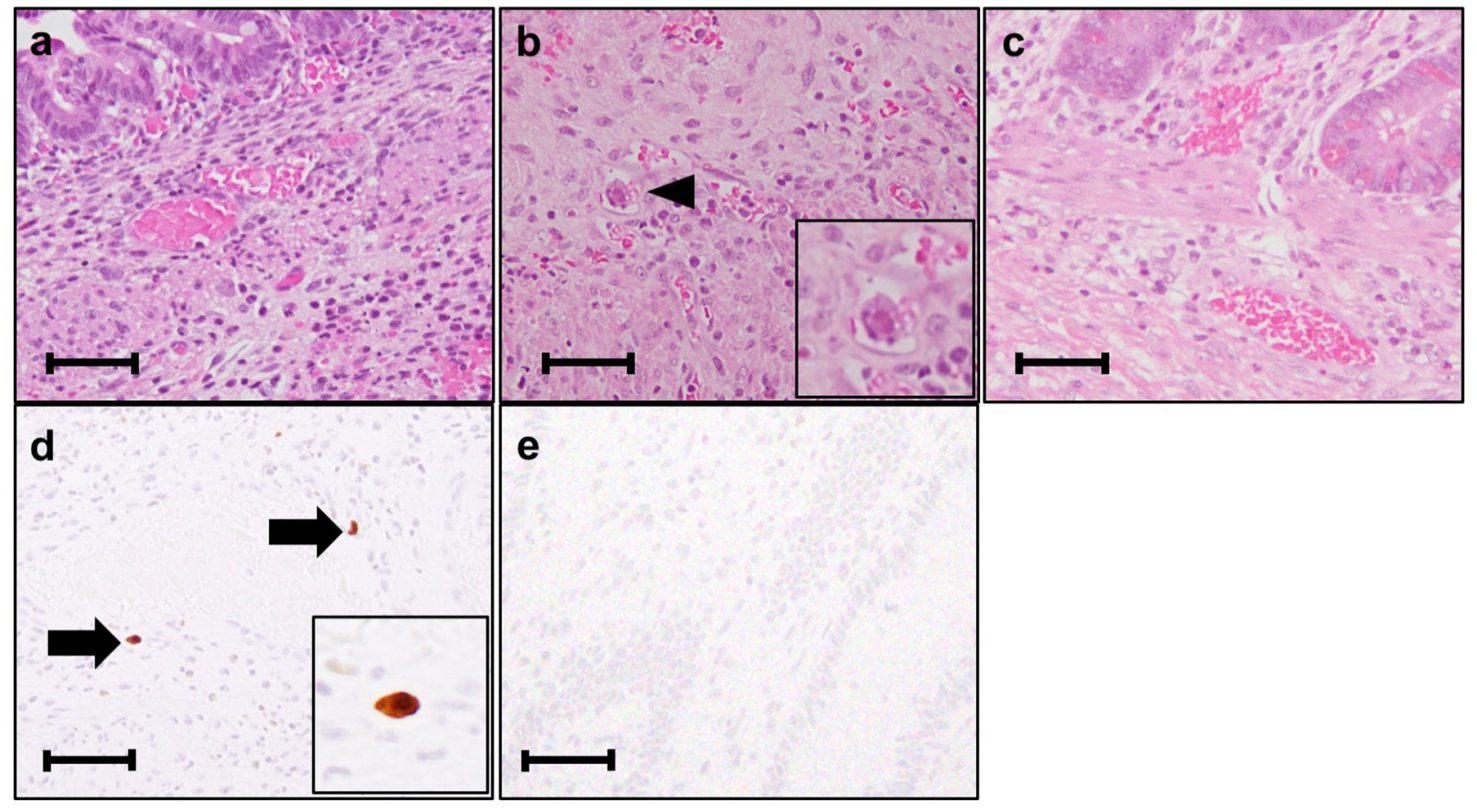
Histological analysis of the intestinal resection specimens. **(a)** Resected ileum at the 1st perforation at day of life (DOL) 8. The vasodilatation and bleeding are observed. (Hematoxylin-eosin stain, original magnification × 400). **(b)** Resected ileum at the 2nd perforation at DOL17. An arrowhead indicates the inclusion body. (Hematoxylin-eosin stain, original magnification × 400). **(c)** Resected ileum at the final ileostomy closure at DOL94. Inclusion body is not identified. (Hematoxylin-eosin stain, original magnification × 400). **(d)** Resected ileum at the 1st perforation at DOL8 immunostained with anti-cytomegalovirus (CMV) antibody. (clone DDG9/CCH2, Dako) Arrows indicate the CMV immunostaining-positive endothelial cells. (original magnification × 400). **(e)** Resected ileum at the final ileostomy closure immunostained with anti-CMV antibody. No CMV positive cells are observed. Each scale bar indicates 50 μm.

A male infant was born at 26 weeks and 5 days of gestational age (GA), with a birth weight of 1,100 g (+ 0.9 SD for GA) from a CMV-seropositive mother. Pregnancy was complicated with uterine contraction for 3 days before spontaneous vaginal delivery, however, the mother's infectious episode during pregnancy was not reported. His Apgar scores were 1 at 1 min and 1 at 5 min. He was initially treated for respiratory distress syndrome, dyspepsia and symptomatic patent ductus arteriosus (PDA). Indomethacin and inotropic support using dopamine and dobutamine were started from day of life (DOL)1. However, indomethacin was not effective and the infant was transferred to our hospital at DOL3 for surgical treatment of PDA. Physical examination on admission was not remarkable for microcephaly (head circumference: 25.0 cm [+ 0.7 SD for GA]), hepatosplenomegaly, rash, jaundice and any neurologic signs. The initial laboratory testing did not show abnormal findings including anemia, thrombocytopenia, elevated liver enzymes or conjugated hyperbilirubinemia (Table 1). Blood coagulation was not remarkable for the infant's prematurity (i.e., prothrombin time: 1,31 INR; fibrinogen: 112 mg/dl; d-dimer: 2.74 μg/ml). Head ultrasound did not reveal any abnormalities such as periventricular calcifications. On the day of admission, he underwent the ligation of PDA and was stabilized hemodynamically. Formula milk feeding was started on DOL4 and then unpasteurized breast milk was added from DOL7. However, dyspepsia persisted and abdominal distention appeared and enteral feeding was stopped at DOL7. Despite being fasted and treated with hyperosmolar enemas from DOL7, the effect was limited. At DOL8, bilious residuals were collected from the nasogastric tube and abdominal radiography showed pneumoperitoneum (Figure 1A). Emergent laparotomy revealed a perforation of the terminal ileum on the mesenteric side, which is opposite to predilection site of NEC, and resection of the perforated site with ileostomy construction was performed. The infant received transfusion of packed red blood cells during the operation at DOL8. Pathological examination of the resected ileum revealed non-specific inflammatory changes which were consistent with enterocolitis and CMV inclusion bodies were not identified (**Figure 3a**). After the operation, dyspepsia persisted and hepatic transaminases were elevated from DOL14 (Figure 2). Abdominal distension appeared again at DOL17 and abdominal radiography showed recurrence of pneumoperitoneum (Figure 1B). He underwent laparotomy and a perforation was found just proximal to the ileostomy. Serum anti-CMV IgG and IgM, that was performed for the differential diagnosis for the elevated hepatic transaminases, were both positive at DOL28. Pathological examination of the second operation, which was reported at DOL30, showed characteristic CMV intranuclear inclusion bodies in the endothelium of lamia propria (Figure 3b). Immunostaining for CMV with the intestinal specimen at the first surgery on DOL8, which was performed retrospectively after the result of pathological examination of the second operation was reported, demonstrated small amount of CMV positive cells (Figure 3d). CMV-DNA was also detected in both blood and urine at DOL28. Moreover, CMV-DNA was detected in the dried umbilical cord, which was obtained within a week from birth and traditionally preserved at the obstetric clinic in Japan as a precious keepsake to parents, suggesting prenatal transmission of CMV from his mother (10).

**Table 1:** Complete Blood Count and Blood Chemistry during the Course of Disease.

**DOL**	**3**	**8**	**17**	**34**	****~**48**	****~**62**	**77**	**146**
Event	Admission	1st perforation	2nd perforation	Before GCV	2 w after GCV	4 w after GCV	6 w after GCV	discharge
Red blood cell (× 10^4^/μl)	452	329	3.75	348	426	335	399	364
Hemoglobin (g/dL)	16.2	11.0	11.8	9.7	12.7	10.0	11.8	9.9
White blood cell/μl	7,330	15,840	13,830	15,570	11,970	4,160	9,780	8,520
Neutrophil/μl	3,518	9,425	7,468	3,970	2,822	832	4,499	2,812
Platelet (× 10^3^/μl)	259	234	89	295	235	344	266	493
AST (U/L)	18	14	103	28	25	37	35	41
ALT (U/L)	3	9	18	22	8	16	21	25
Direct-bilirubin (mg/dL)	1.1	0.8	1.5	0.9	0.7	0.6	0.3	<0.1
Creatinine (mg/dL)	1.1	0.76	0.53	0.41	0.32	0.3	0.26	0.19
CRP (mg/dL)	0.01	0.01	1.35	0.71	0.12	0.25	0.04	0.02

*DOL, day of life; GCV, ganciclovir; AST, aspartate aminotransferase; ALT, alanine aminotransferase; CRP, c-reactive protein*.

Although there are no clinical trials suggesting the benefit of GCV for CMV infections in the preterm neonates (e.g., gestational age ≤ 32 weeks or weighing ≤1,200 g at the onset of treatment) or CMV-associated intestinal disease in the neonates, we chose the treatment with intravenous GCV 12 mg/kg/day for 6 weeks, considering the severity and persistence of the disease and the result of the viral study suggesting the congenital infection of CMV. After starting GCV therapy at DOL34, dyspepsia and abdominal distension resolved and CMV-DNA in the blood was concurrently decreased to lower than detection level by DOL70 (Blood CMV-DNA: 1.3 × 10^7^ [copies/mL] at DOL 28; 3.6 × 10^6^ at DOL 35; 6.2 × 10^4^ at DOL 56; 2.9 × 10^4^ at DOL 63; less than detection level at DOL 70 and 77, Figure 2). No recurrence of gastrointestinal symptoms had occurred thereafter and the ileostomy was closed at 3 months of life. CMV inclusion body or CMV immunoreactivity were not detected in the resection specimen at the final ileostomy closure (Figures 3c,e). During GCV therapy, transient and spontaneously recovered neutropenia was observed around 4 weeks after the initiation of GCV therapy (Table 1). No other acute toxicity of ganciclovir including anemia, thrombocytopenia, liver dysfunction or renal dysfunction was not encountered during and after GCV treatment (Table 1). No abnormal finding with electroencephalography, brain magnetic resonance imaging and auditory brainstem response audiometry were noted before discharge. He was discharged from the hospital at 5 months of life and had been doing well without any neurodevelopmental sequelae noted during the 5-year follow-up.

## Discussion

This case report shows that recurrent intestinal perforations occurred in a CMV-infected premature infant with refractory NEC like symptoms which resolved after a 6-week GCV therapy. Resolution of clinical symptoms (i.e., dyspepsia, abdominal distension, recurrent perforations) and laboratory findings relevant to CMV infection (i.e., CMV-DNA, characteristic CMV inclusion body or CMV antigen in the intestinal pathological specimens) were temporally associated with GCV therapy. Although the role of CMV infection for this disease entity is unclear, our present case suggests that anti-viral treatment may be beneficial for some premature infants with severe gastrointestinal diseases associated with CMV.

The present case was born at 26 weeks' gestation from a CMV-seropositive mother, CMV-DNA was found in the dry preserved umbilical cord obtained within a week from birth (10) and the CMV immunoreactivity in the intestinal resection specimen was detected at the first perforation occurred at DOL8. Although postnatal infection of CMV through the maternal milk given at DOL7 could not be completely excluded, these findings indicates that CMV was most likely transmitted prenatally in this case.

There have been several case reports or case series describing CMV-associated intestinal involvement in preterm infants such as NEC and SIP (6–8, 11). Recent reports have shown that CMV infection may increase vulnerability to secondary bacterial invasion and may also drive proinflammatory immune response, further exacerbating the pathology of NEC and the expression of inflammatory mediators triggered by CMV such as 5-lipoxygenase may drive the inflammation in the bowel and result in exacerbation of the course of NEC. Bonnard et al. also reported that CMV may cause intestinal atresia and perforation when combined with other local factors such as ischemia (12). CMV infection in the gastrointestinal tract is associated with vasculitis of an affected segment and cause perforation in the immunocompromised host or premature or low birth weight infants (13). Thus, although the incidence or the causality of CMV for gastrointestinal involvement in premature infants are not demonstrated to date, there are accumulating findings which support a hypothesis that CMV has an important role in such gastrointestinal disease at least by exacerbating the course of disease. Other risk factor for SIP should be considered as a causative etiology in this case. Extreme prematurity, indomethacin, hydrocortisone or inotropes could be a potential risk factors for SIP in premature neonates (1, 14). The infant received indomethacin from DOL1 to DOL2 and inotropes including dopamine and dobutamine from DOL1 prior to the first SIP but not hydrocortisone. Although CMV was presumably transmitted prenatally in this case, the infant lacked marked symptoms for CMV infection in the first week of life, but exhibited the elevation of liver enzymes at DOL14 prior to the second SIP and CMV intranuclear inclusion bodies were found in the intestinal resection specimen at DOL17. This course of events suggests that persistent abdominal symptoms occurred around the second SIP at DOL17 may be largely attributable to CMV, while the first SIP at DOL8 may be rather attributable to other risk factors to SIP (e.g., indomethacin use for PDA, extreme prematurity) than CMV. Although extreme prematurity and the other risk factors for SIP may have all contributed to the disease course, CMV infection may contribute, at least to the persistency of the abdominal symptoms following the SIP and GCV may impede progressive and refractory, presumably otherwise uncontrollable, gastrointestinal symptoms in this case.

Among the causative etiology of severe gastrointestinal complication in premature infants (e.g., prematurity, ischemia, indomethacin for PDA), CMV infection is regarded as rare (13) and generally not included in the differential diagnosis of gastrointestinal disease in premature newborns, however recent study showed that CMV infection was highly prevalent in surgical intestinal specimen from infants with NEC or SIP and may exacerbate the course of NEC (11, 15). Since definitive diagnosis of CMV infection in the intestine requires obtaining bowel tissue, there may be underdiagnosis of CMV-associated gastrointestinal disease in premature infants (11). These observations indicate that CMV should be considered for the underlying etiology for the exacerbation of gastrointestinal disease in premature infants and anti-CMV therapy may be a supplemental treatment option which might impede progression of gastrointestinal disease in some premature infants.

Although there have been a few cases with gastrointestinal disease in CMV-infected neonates treated with GCV (Supplemental Table 1) (7, 8, 12, 16–18), the benefit of GCV therapy for this condition has not been fully demonstrated. Tengsupakul et al. reported that treatment with GCV resulted in clinical improvement and disappearance of viremia in a preterm infant with CMV-associated NEC (8). In the presented case, we confirmed, for the first time to our knowledge, the disappearance of CMV inclusion body and CMV-antigen in the affected intestine as well as CMV-DNA in the blood along with the resolution of abdominal symptoms following recurrent ileal perforations after the GCV treatment. In addition to the symptomatic infants with congenital CMV infection, GCV has been used for a variety of serious perinatal or neonatal illnesses caused by congenital CMV infections, including non-immune fetal hydrops, myocarditis, pneumonitis, hepatitis and intrahepatic cholestasis (19–23). The use of GCV therapy for congenital CMV infection and disease has generally been reported to be safe and well-tolerated, and has appeared to be useful in ameliorating the severity of focal, end-organ disease (21–24). However, there are also reports showing the limited effect of GCV in infants with fulminant course of CMV infection (19, 20, 25). The main toxicity related to GCV treatment for congenital CMV infection was the development of a clinically significant neutropenia (63% of treated patients vs. 21% in the non-treated group) (9), as was observed in the present case. It should be also noted that GCV therapy has the potential for long-term gonadal toxicity (26) or carcinogenicity (27). Although the present case did not have irreversible acute toxicities of GCV and had no neurodevelopmental sequelae at the corrected age of 5 years, further follow-up is needed for comprehensive long-term development and late adverse effects.

In the course of severe CMV-associated neonatal disease, GCV treatment was initiated relatively late in cases described in the previous reports as well as our report, particularly in preterm infants (20–22). In the present case, because the infant initially lacked typical clinical findings relevant to congenital CMV infection, the role of CMV as an etiology for the SIP was not suspected until the result of pathological examination (i.e., presence of CMV inclusion body) was reported on DOL30. Since delay of diagnosis of CMV infection and initiation of treatment may limit the efficacy of anti-viral therapy in neonates with such end-organ diseases, particularly in which CMV is regarded as a rare causative etiology (20), development and implementation of screening programs for identification of congenital CMV infection, using maternal serologic testing and potentially newborn blood spots obtained for the metabolic screen, could aid in identifying CMV as a causative etiology for severe end-organ diseases and improving outcome in the affected infants (5, 8, 28). Since postnatal infection of CMV from fresh breast milk also cause such severe illnesses in neonates (4, 6, 29), the technique including pasteurization of breast milk from CMV-seropositive mother may also be an option for high risk infants as extremely preterm infants considering the potential benefit of fresh human milk vs. the risk of CMV transmission (30, 31). Finally, the ultimate control of CMV disease in newborns will most likely depend upon the development of an effective vaccine, which, if administered to young women prior to their child-bearing years, could reduce the burden associated with this important public health problem even in premature infants (32).

Collectively, our present observation warrants further studies to determine the pathogenic role as well as the accurate incidence of CMV infection and the role of anti-viral therapy in preterm neonates with severe gastrointestinal disease.

## Data Availability Statement

The original contributions presented in the study are included in the article/Supplementary Material, further inquiries can be directed to the corresponding author/s.

## Ethics Statement

Written informed consent was obtained from the individual(s), and minor(s)' legal guardian/next of kin, for the publication of any potentially identifiable images or data included in this article.

## Author Contributions

MM, HS, NY, HO, RH, KS, HT, YM, YKom, and MH managed the patient, contributed to the conception of the study, and drafted the manuscript. KT and TI managed the mother and performed the viral studies, contributed to the conception of the study, and drafted the manuscript. KMat, YKoi, KO, MI, and KU performed the surgeries, contributed to the conception of the study, and drafted the manuscript. HI performed the pathological examinations and drafted the manuscript. KMar, YM, YKom, TI, and MH critically reviewed the manuscript. All authors read and approved the final manuscript.

## Conflict of Interest

The authors declare that the research was conducted in the absence of any commercial or financial relationships that could be construed as a potential conflict of interest.

## Abbreviations

CMV: cytomegalovirus
GA: gestational age
DOL: day of life
GCV: ganciclovir
NEC: necrotizing enterocolitis
PDA: patent ductus arteriosus
SIP: spontaneous intestinal perforation.
