# Supplementary material for: Refractory Ileal Perforations in a Cytomegalovirus-Infected Premature Neonate Resolved After Ganciclovir Therapy
Source: Front Pediatr. 2020 Jul 14;8:352. doi: 10.3389/fped.2020.00352 (PMC7372912; doi:10.3389/fped.2020.00352)
Supplement: Supplementary file 1 [file Table_1.docx]

**Supplemental Table 1**

**Neonates with CMV-associated Gastrointestinal Disease Treated by Ganciclovir**

| GA, week | BW, g | Time and symptoms at onset  Time (DOL), symptom | Breast  feeding | Diagnosis of CMV infection | Diagnosis of CMV-gastrointestinal disease | Anti-viral therapy, duration | Outcome | Ref. |
| --- | --- | --- | --- | --- | --- | --- | --- | --- |
| 24 | **550** | **DOL25,**  **Diarrhea**  **Abdominal distension**  **Pleural effusions** | **yes** | **Urine; CMV detection of early antigen fluorescent foci test+** | **n.a.** | **GCV, 13days** | **Resolved** | **16** |
| 24 | **800** | **DOL26,**  **Abdominal distention** | **yes** | **Blood; PCR+** | **Pathology**  **(CMV inclusion bodies, IHC)** | **GCV, 3weeks** | **Resolved** | **8** |
| 26 | **864** | **DOL19,**  **Tachycardia**  **Abdominal distension** | **yes** | **Blood; IgG-, IgM+, PCR+**  **Urine; culture+** | **Pathology**  **(IHC)** | **GCV, 3weeks** | **Resolved** | **7** |
| 37 | **2490** | **n.a.,**  **Abdominal distention**  **Biliary vomiting** | **yes** | **Blood; IgM+** | **Pathology**  **(CMV inclusion bodies)** | **GCV, 6weeks** | **Resolved** | **17** |
| 39 | **3290** | **DOL1,**  **n.a.** | **n.a.** | **Blood; IgM+**  **Urine; culture+** | **Pathology**  **(IHC)** | **GCV, 5weeks** | **n.a.** | **12** |
| term | **1600** | **DOL15,**  **Neck retraction, tachypnea, jaundice, loose stools** | **n.a.** | **Blood; IgM+**  **Urine; PCR+, culture+** | **PCR with intestinal biopsy** | **GCV, 6weeks** | **Resolved** | **18** |

Abbreviations: CMV, cytomegalovirus; GCV, ganciclovir; Ref, reference; GA, gestational age; BW, birth weight; DOL, day of life; n.a., not available; IHC, immunohistochemistry
